# Poly (3, 4-ethylene dioxythiophene) Supported Palladium Catalyst prepared by Galvanic Replacement Reaction for Methanol Tolerant Oxygen Reduction

**DOI:** 10.1038/s41598-019-55688-5

**Published:** 2019-12-16

**Authors:** Pandia Rajathi M, Sheela Berchmans

**Affiliations:** 1 EEC Division, CSIR-Central Electrochemical Research Institute Karaikudi, 630003 Tamil Nadu, India; 2 Academy of scientific and innovative research (AcSIR), New Delhi, India

**Keywords:** Fuel cells, Fuel cells

## Abstract

Herein, we propose a facile electrochemical approach for the synthesis of Pd loaded poly 3, 4-ethylenedioxythiophene (PEDOT) electrodeposited on glassy carbon electrode (GCE) resulting in high surface area. The catalyst preparation is initiated with EDOT polymerization on GCE surface by electrochemical potential cycling method, followed by the electrodeposition of Cu from a 2 mM solution of CuSO_4_ in 0.1 M NaClO_4_ at a constant potential of +0.34 V *vs*. SHE in the form of Cu nanocubes on the PEDOT surface. Pd-PEDOT catalyst was then prepared by the partial substitution of copper by galvanic displacement with various concentrations of PdCl_2_. The prepared Pd/PEDOT electrocatalyst is found to be methanol resistant indicating its usefulness as fuel cell cathode. The prepared catalyst supports two electron transfer of oxygen reduction reaction in 0.5 M H_2_SO_4_. The effects of Pd and Cu contents and the quantity of PEDOT, mass and specific activities were studied. At a relatively low Pd loading of 0.57 ng*/cm*^2^, the Pd/PEDOT should be a cost-effective alternative cathode catalyst for direct methanol fuel cells, DMFCs. This work explains the usefulness of PEDOT as good catalyst supporting material which is prepared by an eco-friendly electrochemical route.

## Introduction

The global demand for electricity generation and its vast usage has motivated the researchers to find alternative renewable energy solutions. Electrochemical conversion of chemical energy into electrical energy is more advantageous than heat engine based energy conversion where Carnot’s theorem poses limitation. The electrochemical energy conversion devices that are of high significance are fuel cells, electrolysers, batteries, supercapacitors and redox flow batteries. There is a dire need for electrochemical energy conversion devices as the demand for off grid portable devices and electric vehicles increases. In a fuel cell the conversion of chemical to electrical energy happens by anodic oxidation of fuel resulting in the liberation of electrons which flows through the exterior circuit and arrive at the cathode wherein most of the cases oxygen reduction occurs. Small molecules like hydrogen, methanol, ethanol, formic acid etc., act as fuels in fuel cells^1^. Fuel cell driven electrochemical engines would offer clean source of energy and such engines would be noise free, vibration free and devoid of harmful gaseous emissions. Pt is the best catalyst ever known for fuel cell applications. The poor kinetics of oxidation of fuels at the anode and low abundance combined with the exorbitant cost of Pt make the cost of the device very high. Hence researchers are focusing on alternate catalysts with enhanced performance Also, investigations on the electrochemical oxidation of small molecules like methanol, formic acid are on the increase besides hydrogen^2–7^.For many of the applications including fuel cells in which Pt reigns supremacy, the current trend is to make use of alloy of noble metals with non-noble metals^8,9^. Such alloy catalysts are helpful in reducing the overall cost of the catalyst and exhibit much better performance and high selectivity^10–14^. Nowadays, metallic platinum (Pt) and Pt-based alloys are considered widely popular and superior electrocatalysts for cathodic reaction (oxygen reduction, ORR) in fuel cell applications, metal air batteries, chloralkali electrolysis, water electrolysis, metal corrosion processes etc., In addition to their low abundance and exorbitant cost of Pt based catalysts, they also suffer from low stability, and their susceptibility to fuel (e.g., methanol) poisoning effects. Therefore, we are faced with the big challenge of development of Pt-free electrocatalysts for ORR with improved catalytic performance and durability. Owing to the similarity of Palladium to Pt in terms of crystal structure, electronic configuration and its position at the apex of the volcano plot for ORR activity of pure metals as published by Norskov *et al*., Pd has emerged as a suitable alternative cathode catalyst for fuel cell applications^15^. The high availability and low cost of Pd are the additional advantageous features in support of Pd being a suitable cathode catalyst. It has been shown that Pd-based electro catalysts exhibit good ORR activity in alkaline media. Also it is known that Pd-based electrocatalysts exhibit poor catalytic activity towards ORR in acidic media when compared to Pt-based electrocatalysts. In order to prevent leaching of metal/alloy nanoparticle catalysts from the electrode surface supporting matrices need to be incorporated on the electrode surface. Supporting matrices provide additional advantages such as (1) improved surface area caused by better distribution of nanoparticles (2) enhanced diffusion of electroactive species through porous supports (3) promoting catalytic activity by increasing electron transfer rates/decreasing Fermi level of catalysts. Usually carbonaceous materials, metal oxides and polymers are used as support materials for catalysts. Although carbonaceous materials are traditionally used as supporting materials, the problems associated due to carbon corrosion necessitate the quest for alternate supports for catalysts. Conducting polymers belong to the class of smart materials with promising and novel applications such as thin film field effect transistors, polymeric light emitting diodes, magnetic shielding materials, sensor technology and so on^16–19^. Conducting polymers are ideal for a broad range of applications because of their special properties, such as high conductivity, stability in air and ease of preparation. Also they can act as a supporting matrix for a dispersed catalyst providing a large surface area that is essential for effective electrocatalysis. In this investigation, the conducting polymer aids high catalytic activity of the nano sized catalytic material. Generally, the combination of chemically prepared polymer and Pd nanoparticles leads to a material with unique properties suitable for multifarious applications and they are predicted as excellent catalysts for oxygen reduction in alkaline medium^20^. In order to avoid aggregation, poor distribution and leaching of naked Pd NPs, conducting polymers provide an ideal choice for catalyst supports. A new strategy of galvanic displacement is adopted in this work to obtain nano sized and well dispersed Pd NPs supported on PEDOT matrix without stabilizers. The literature reports on Pd based ORR catalysts reveal that only carbonaceous materials, metal oxides, chalcogenides and metal carbides have been evaluated so far as catalyst supports^21^. Further only few catalyst preparations involve electrochemical methods. Hence, this paper discusses the first attempt made towards the evaluation of PEDOT as suitable catalyst supports for Pd where the nanostructured Pd catalyst has been prepared by electrodeposition of Cu followed by galvanic replacement of Pd. It is well known that Pd can galvanically displace Cu^22,23^. Motivated by the preceding studies, we planned a strategy of initially depositing Cu on PEDOT matrix and then impregnating Pd NPs by galvanic displacement phenomenon between Pd ions and Cu. Pd has higher formal electrode potential then Cu and therefore Pd replaces the Cu from Cu nanocubes on PEDOT surface and thus formed Pd NPs which resembled shrimp like structure. Moreover, the electrochemical data indicate that the Pd/PEDOT catalyst simultaneously exhibits better activity for oxygen reduction reaction in acid medium along with methanol tolerance and can be used as cathode in methanol fuel cell and for hydrogen peroxide preparation.

## Experimental Methods

### Reagents

Analytical grade chemicals were used without further purification for all the experiments. The monomer 3,4-ethylenedioxythiophene (EDOT, TCI), tetrabutyl ammonium perchlorate (TBAClO_4_, Fluka), acetonitrile (E-Merck), copper sulphate (Hi Media), sodium perchlorate (Sigma-Aldrich), sulphuric acid (E-Merck), palladium (II) chloride (sigma-Aldrich) and acetonitrile (ACN) were pure and used as received.

### Preparation of PEDOT on Glassy carbon, GC surface

The GC electrode was subjected to pre-treatment by polishing on alumina slurry using emery cloth followed by sonication in acetone + water mixture and water respectively. After the pre-treatment, the cleaned GC electrode was dried under vacuum. Then the dried GC electrode was investigated by cyclic voltammetry in the scan range from 0.0 V to 1.4 V *vs*. SHE. Then the electrode was washed with milli-Q water and allowed to dry. The 20 mM of EDOT monomer was prepared in 50 ml of acetonitrile and 50 mM of tetrabutyl ammonium perchlorate and was used for electropolymerization. The EDOT was electrochemically polymerised on GC electrode by cyclic voltammetry between the potential limits −0.65 V and 1.25 V *vs*. Ag^+^ at a scan rate of 0.05 V/s. A smooth thin blue coloured film was formed in 10 cycles when an Ag^+^ and platinum foil acted as the pseudo reference and counter electrodes for the electrochemical cycling experiments. The PEDOT/GC electrode was then rinsed with ACN solution, to remove the excess of PEDOT^24^.

### Electrochemical deposition of Cu NPs on PEDOT/GC

The PEDOT/GC electrode was investigated in an aqueous solution of 0.1 M NaClO_4_ in the potential range from −0.65 V to 1.25 V *vs* Ag^+^ at a scan rate of 0.05 V/*s*. Cu was electrochemically deposited on PEDOT/GC electrode using a 2 mM solution of CuSO_4_ in 0.1 M NaClO_4_ at a constant potential of +0.34 V *vs*. SHE for 120 *s*. Then the electrodeposited Cu on PEDOT electrode was washed with milli-Q water. The Cu modified PEDOT/GC electrode was examined by cyclic voltammetry in the scan range from 0.0 V to 1.25 V at a scan rate of 0.05 V/*s vs*. SHE.

### Galvanic replacement of copper by Palladium

The Cu/PEDOT electrode was subjected to the galvanic replacement reaction and the Cu NPs were replaced by Pd NPs. The Cu/PEDOT electrode was kept immersed in different concentrations (0.1, 0.5, 1, 2, and 3 mM) of Palladium chloride (PdCl_2_) solutions for 30 minutes without any disturbance. After 30 minutes, the electrode was washed with Milli-Q water and was then allowed to dry for few minutes.

### Electrochemical characterisation

The electropolymerization was carried out as described by us earlier^24^ and as mentioned in section 2.2. Briefly electropolymerisation was carried out on a well-polished and cleaned GC electrode from a 0.02 M EDOT (3, 4-ethylenedioxythiophene monomer) dissolved in 100 ml of a 0.05 M solution of TBAClO_4_ (tetrabutyl ammonium perchlorate) in acetonitrile. Potential cycling (20 segments) was conducted between the potentials −0.65 V and +1.25 V *vs*. an Ag^+^/Ag reference electrode. A Pt foil was used as the counter electrode. Subsequent to polymerization, the GC electrode was rinsed completely with acetonitrile. Cu was electrodeposited on GC/PEDOT in the form of nanoparticles (NPs) at a constant potential of +0.34 V *vs*. SHE (120 s of deposition) from a 0.002 M solution of CuSO_4_ in 0.1 M NaClO_4_ solution. The Milli-Q water (ρ = 18.2 MΩ cm) was used to prepare the electrolyte solution. The mass of electrodeposited Cu was calculated by cyclic voltammetry (CV). The Cu NPs were partially replaced by Pd by galvanic displacement reaction (GDR) by immersing the Cu NP modified PEDOT/GC electrode for 30 min in PdCl_2_ solutions of different concentrations. The galvanic replacement phenomenon was confirmed by CV in 0.5 M of H_2_SO_4_. The charge, mass and electroactive surface area (EASA) of Pd-Cu PEDOT/GC were calculated from CV data. The same procedure was repeated for different concentrations of PdCl_2_. Thus fabricated Pd/PEDOT/GC electrocatalyst was used to study the methanol tolerant oxygen reduction reaction. The potential values are stated with reference to SHE or Ag^+^/Ag in the graphs presented in this manuscript.

A CHI 1000 A (Electro Analytical System) was employed for electropolymerisation of EDOT by sequential potential cycling, controlled potential deposition of Cu and for the estimation of Pd content by CV. Oxygen reduction reaction was examined by rotating disk electrode (RDE) and rotating ring disk electrode (RRDE) supplied by Pine instrument. Morphological details were estimated by various techniques as described us earlier^24^. The information related to the structure and size of the Pd/PEDOT/GC and Cu/PEDOT/GC catalysts were obtained by transmission electron microscopy (TEM) (FEI Tecnai 20 G2). X-ray diffraction (XRD) studies were conducted by preparing the nanostructured catalyst on a ITO substrate, by following the same method with a Bruker D8 Advance X-ray diffractometer with Ni-filtered Cu K_α_ radiation (λ = 1.5406 Å) at the scan rate of 3°/min in steps of 0.05°. The percentage of Pd and Cu on PEDOT was investigated by EDAX (Hitachi S-3000H scanning electron microscope) and the distribution of Cu and Pd on PEDOT was examined by a Carl Zeiss field-emission scanning electron microscope (SEM) (Supra 55 VP). The chemical states of the elements were determined by a Multilab 2000 Thermo Scientific X-ray photoelectron spectrophotometer (XPS) using a twin anode X-ray source (Mg K_α_ radiation, 1253.6 eV). The XPS spectra were curve fitted and de-convoluted with XPS peak 4.1 software. The topography image was recorded by scanning probe microscopy Agilent technology (5500 series) and the image was processed by pico image basic software.

## Results and Discussion

### Spectral characterizations

#### FT-IR spectroscopy for Pd/PEDOT

**Figure 1:**
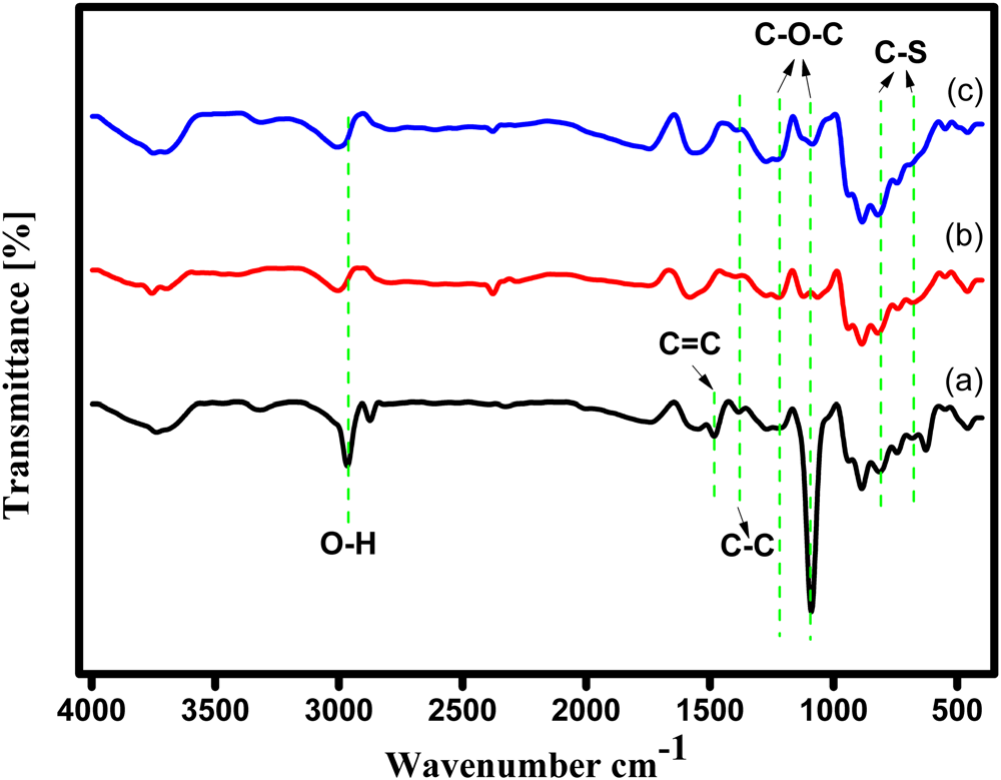
FT-IR Spectra for (**a**) polymerized EDOT monomer (PEDOT), (**b**) electro deposited Cu-PEDOT, and (**c**) galvanically replaced Pd on Cu-PEDOT.

The prepared catalyst has been examined by FTIR during every stage of electrode modification. Figure 1 depicts the FT-IR of the composite material which reveals the bonding interaction and the vibration modes in every functional group before and after doping Cu and Pd. Figure 1a shows the vibrational bands at 1556 and 1386 cm^−1^ corresponding to the formation of C=C and C-C of the quinodial structure of thiophene ring, respectively.

The vibrational bands at 1209 and 1089 cm^−1^ are ascribed to the C-O-C stretching vibrations in ethylenedioxy group. The C-S band stretching in thiophene ring appeared at the absorption bands at 813 and 678 cm^−1^. The vibrational bands at 939 cm^−1^ is due to the ethylene dioxy ring deformation mode. The Fig. 1b corresponding to Cu NPs functionalized polymer matrix, reveals that the peak corresponding to dioxy group is missing which shows that the Cu is complexed at the oxygen site. A marginal change is observed in the C-S region, indicating only insignificant complexation of Cu atoms at the Sulphur site. In the case of the Pd NPs decorated PEDOT/GCE the intensity of the peaks increased due to the doping of Pd NPs as shown in Fig. 1c.

#### X-ray diffraction analysis

**Figure 2:**
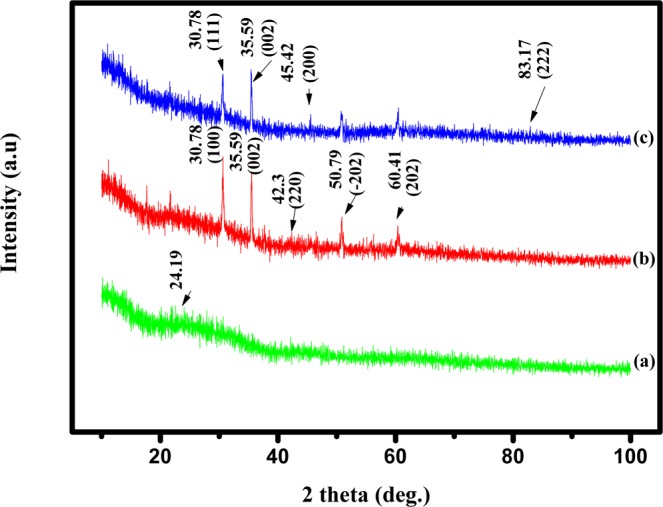
X-ray diffraction pattern for (**a**) electro polymerized EDOT monomer (PEDOT), (**b**) electrodeposited Cu on PEDOT under constant potential, and (**c**) galvanically replaced Pd on Cu-PEDOT.

The XRD spectrum shown in Fig. 2a reveals a peak at a 2*θ* value of 24.19° which confirms the electropolymerization of EDOT and corresponds to planar chain stacking. The experimental XRD peaks of Cu-PEDOT are obtained at 30.78°, 35.59°, 42.3°, 50.79° and 60.41°. It denotes the electrochemical deposition of the copper nanoparticles (Fig. 2b) on PEDOT matrix. The observed peaks correspond to (100), (002), (220), (−202), and (202) crystal planes respectively, confirming the cubic structure of Cu (JCPDS 00-003-0892). The Cu is replaced by Pd NPs via galvanic replacement reaction in 30 minutes. The as prepared Pd/PEDOT/GC electrode (Fig. 2c) shows the peaks of palladium at 30.78°, 35.59°, 45.42°, and 83.17° which signify (111), (002), (200), and (222) crystal planes (JCPDS 01-087-0637).

#### X-ray Photoelectron Spectroscopy

**Figure 3:**
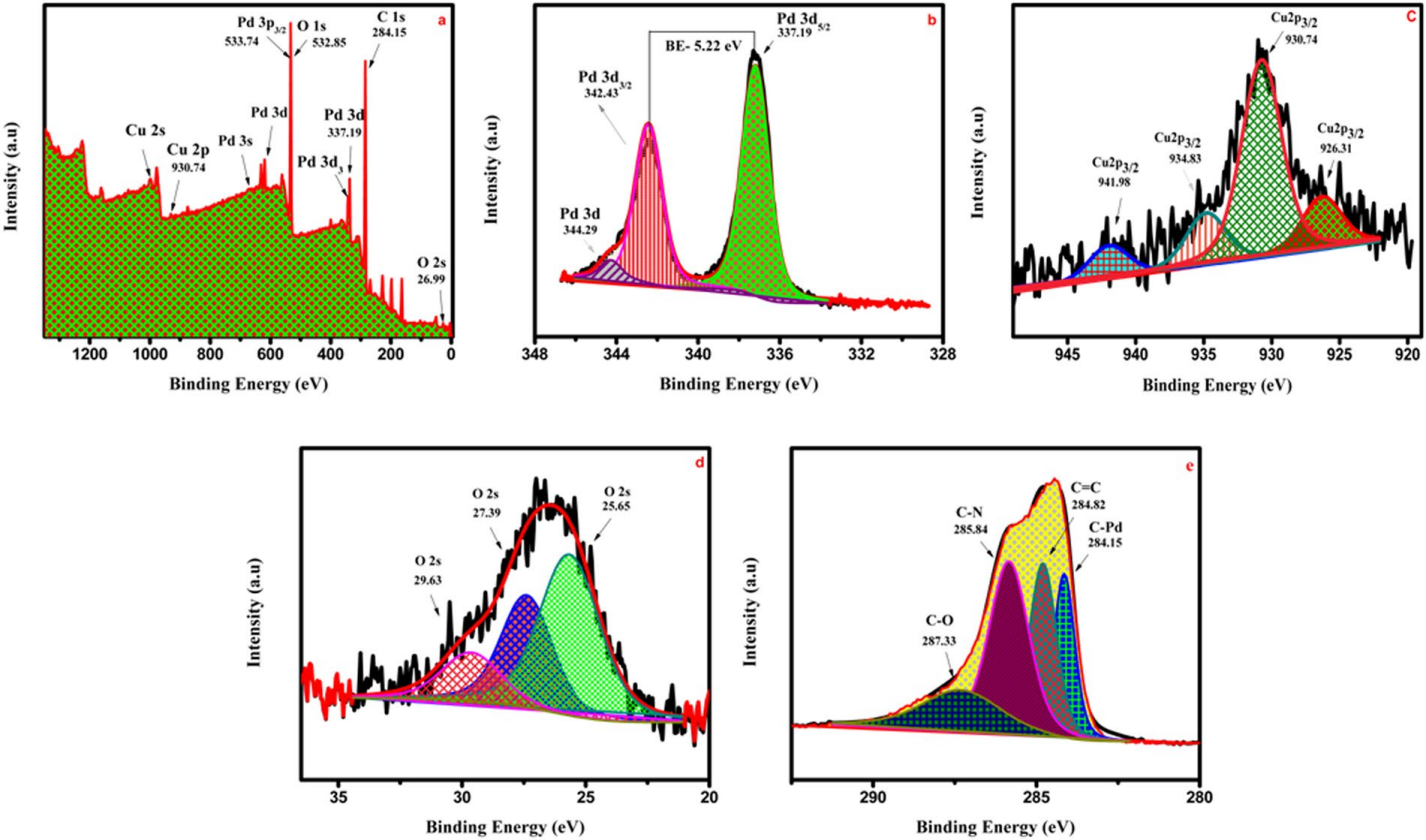
XPS analysis for electrochemically synthesised Pd/PEDOT catalyst, (**a**) survey spectrum showing characteristic photoemission of each element in the sample, (**b**) Pd 3d_5/2_ and Pd 3d_3/2_ spectral line, (**c)** Cu 2p_3/2_ spectral line, (**d**) O 2 s spectral line, and (**e**) C 1 s spectral line.

The chemical composition of Pd/PEDOT is determined by XPS analysis. The composite catalyst shows the presence of the elements Pd, Cu, O, and C. The Gaussian-Lorentzian fitting has been used for deconvolution of Pd 3d, Cu 2p, O 2 s, and C 1 s, respectively. Figure 3a depicts the survey spectrum of the prepared catalyst where core level photo emissions for even trace level impurities are also detectable. The O 1 s appears at approximately 532.85 eV but Pd 3p_3/2_ is also present in the same region. Hence O 2 S peaks are taken into consideration. The high resolution profile of Pd 3d depicted in Fig. 3b shows the pair of asymmetric peaks constituting the Pd 3d signal. The binding energies (BEs) of Pd 3d_3/2_ (342.43 eV) are 5.22 eV lower than those of Pd 3d_5/2_ (337.19 eV) for each doublet^25^. The intense doublet peaks belong to Pd (0) and the weak peaks are attributed to Pd (II) species, such as Pd (OH) _2_ and PdO.

The peak of Cu can be clearly seen at 930.74 eV in the high-resolution spectrum of Cu in Fig. 3c, which indicates distinct distributions of four Cu valence states; that is, the signals observed at 926.31 eV, 930.74 eV, 934.83 eV, and 941.98 eV correspond to Cu^+^, Cu^2+^, Cu(OH)_2_ and Cu respectively. The feature at 930.74 eV can be due to Cu^+^ or metallic Cu. The C 1 s bond can be deconvoluted into three bands at 284.6 eV, 285.9 eV and 287.2 eV, which can be assigned to C=C, C-N (C=N) and C-O (C=O), respectively.

### Morphological characterizations

#### Field Emission Scanning Electron Microscopy (FESEM)

**Figure 4:**
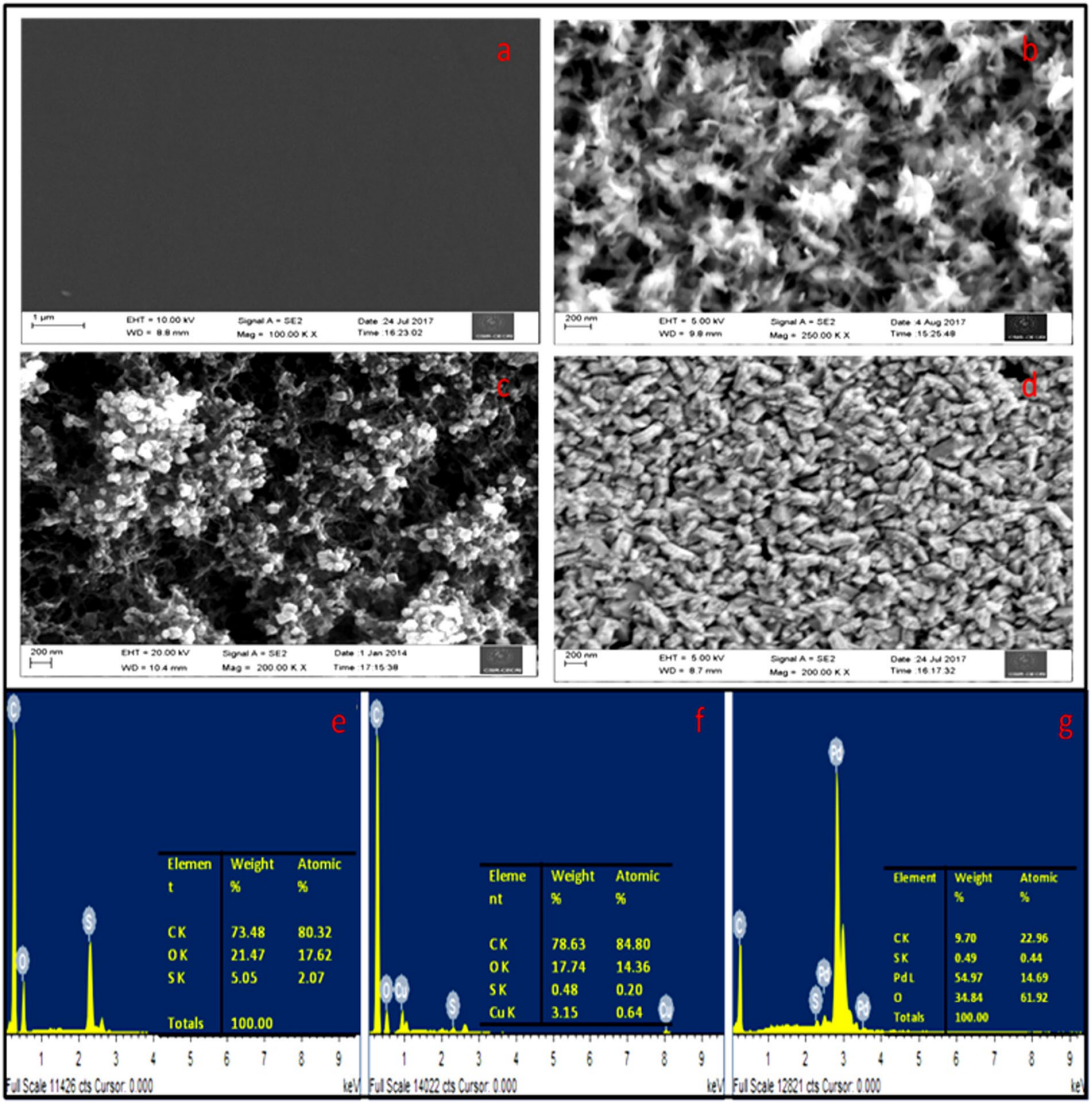
The morphological study of the electrode: FE-SEM images of (**a**) glassy carbon electrode, (**b**) PEDOT(**c**) Cu/PEDOT, and (**d**) Pd/PEDOT. FESEM-EDAX image of (**e**) PEDOT, (**f**) Cu/PEDOT and (**g**) Pd/PEDOT.

The morphologies of (a) GC, (b) PEDOT, (c) Cu-PEDOT, and (d) Pd/PEDOT were characterised by FESEM. The cleaned GC (Fig. 4a) surface indicates the absence of catalyst and the electropolymerized EDOT (PEDOT) on GC (Fig. 4b) surface reveals the 3D porous network structure of PEDOT. The electrochemically deposited Cu on PEDOT (Fig. 4c) resembles cube like structure and the formation of Cu cubes strongly depends on the time, concentration and number of cycles during electrochemical PEDOT deposition. The size of the Cu nano cubes varies from 63 to 152 nm. The Pd/PEDOT/GC (Fig. 4d) prepared by galvanic replacement reaction of Cu by Pd, shows mosaic pattern resembling shrimp fish. Closely placed spherical Pd NPs knitted in the shape of shrimp fish is observed in the case of Pd/PEDOT/GC. It could be observed that Pd replaces the top layer of Cu atoms on Cu nanocubes on PEDOT matrix and takes the shape of nanorods.

The chemical composition of prepared bimetallic catalyst and their weight percentage has been estimated by FESEM-EDAX analysis and it reveals the presence of C, S, and O in the polymer backbone on GC (Fig. 4e–g) and the atomic percentage.

#### Transmission electron microscopy (TEM) analysis

**Figure 5:**
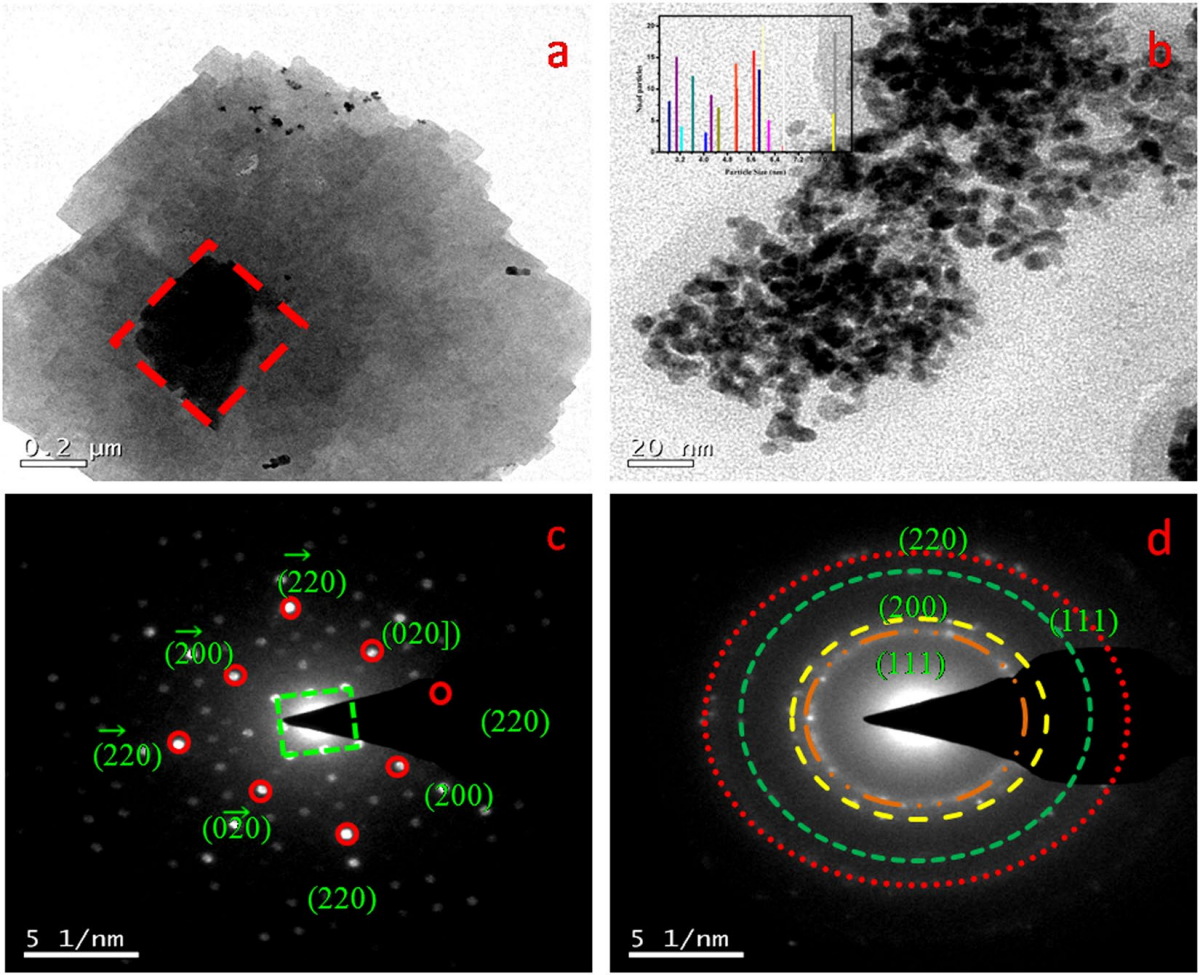
TEM analysis for (**a**) the Cu nanocubes formation on PEDOT surface (**b**) Pd nanoparticles agglomeration on Cu nanocubes on PEDOT, SAED pattern acquired from the irradiated substrate depicting the presence of (**c**) Cu nanocube on PEDOT, and (**d**) Pd NPs on Cu-PEDOT.

Morphological textures of Cu-PEDOT and Pd/PEDOT were investigated by TEM analysis. The Cu-PEDOT depicted in Fig. 5a shows the three dimensional structure of Cu nanocubes on PEDOT matrix, and Pd nanoparticles are displayed in Fig. 5b. The formation of tiny sphere shaped Pd nanoparticles have the size of 2.8 to 8.5 nm range as depicted in Fig. 5b (inset image). The agglomerated Pd nanoparticles stretch over a length of 200 nm resembling a shrimp and it reveals the homogeneous distribution of Pd NPs on Cu/PEDOT matrix.

**Figure 6:**
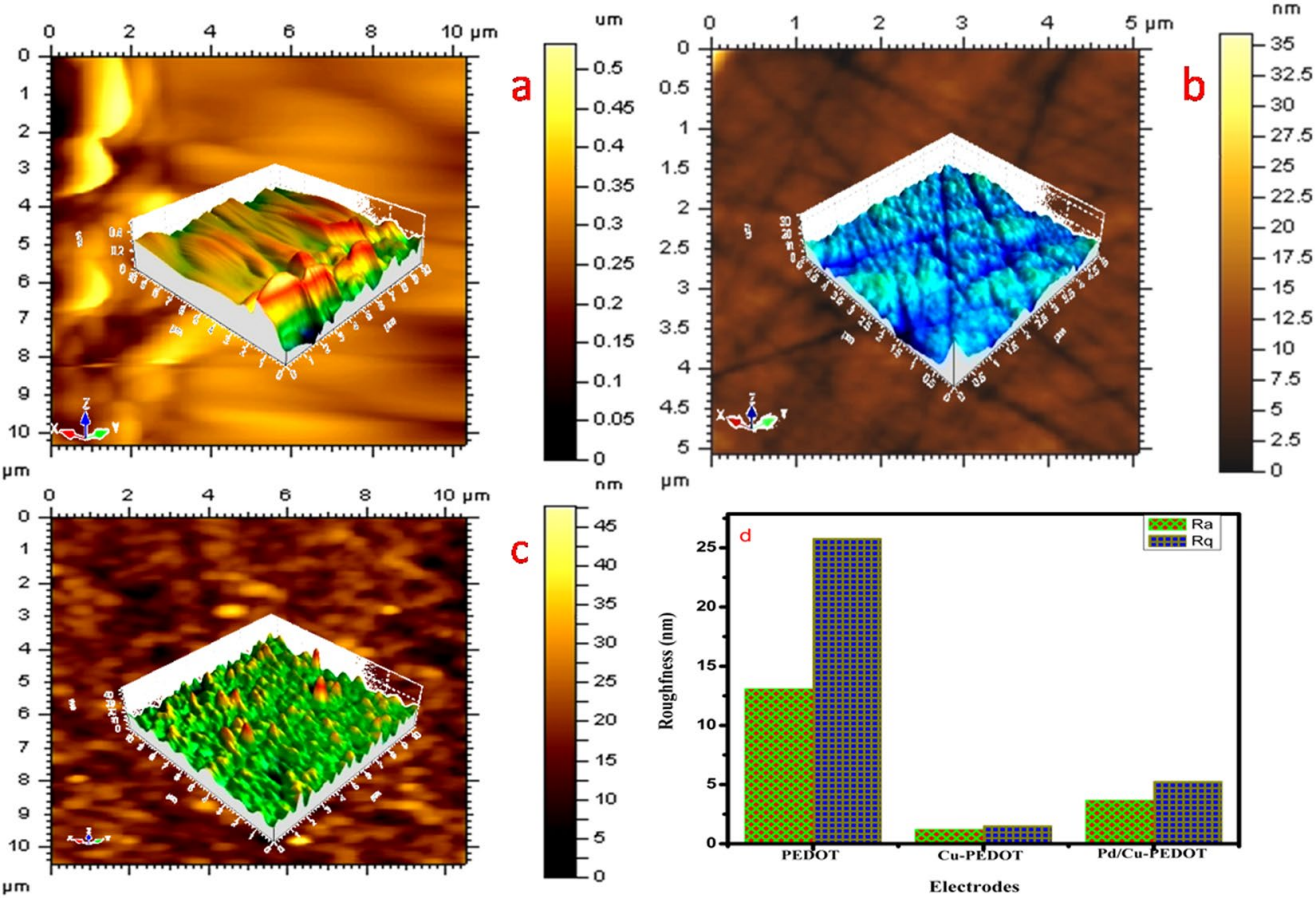
AFM images of glassy carbon modified electrodes at different stages of modification: (**a**) PEDOT, (**b**) Cu/PEDOT, (**c**) Pd/PEDOT, (**d**) root mean square and average roughness of the respective electrodes.

The TEM-SAED pattern depicted in Fig. 5c shows the cubic diffraction of Cu on PEDOT with (220), (020), (200), (−220), (−2–20), (−200), and (0–20) planes of Cu. The agglomerated polynomial Pd pattern (Fig. 6d) with a homogenous distribution of Pd nanoparticles is clearly displayed as a ring arising from the diffraction pattern of (111), (200), and (220) planes of Pd respectively.

#### Atomic force microscopy (AFM) analysis

Surface morphology, thickness and roughness of the electrodes have been studied by AFM analysis. The topography of the modified electrode surface shown in Fig. 6a displays the electrochemical intercalation of PEDOT with high surface roughness that can act as a gateway for the allocation of Cu^2+^ions (Fig. 6b). The electrodeposition of Cu on PEDOT has less roughness factor compared to PEDOT. The galvanically replaced Pd/PEDOT shown in Fig. 6c shows relatively a high surface roughness (*Ra*) and root mean square (*Rq*) compared to Cu-PEDOT. The increased surface roughness shows the adherence of Pd on Cu revealing the union of two surfaces.

### Electrochemical studies

#### Electrochemical characterization of PEDOT on GC

**Figure 7:**
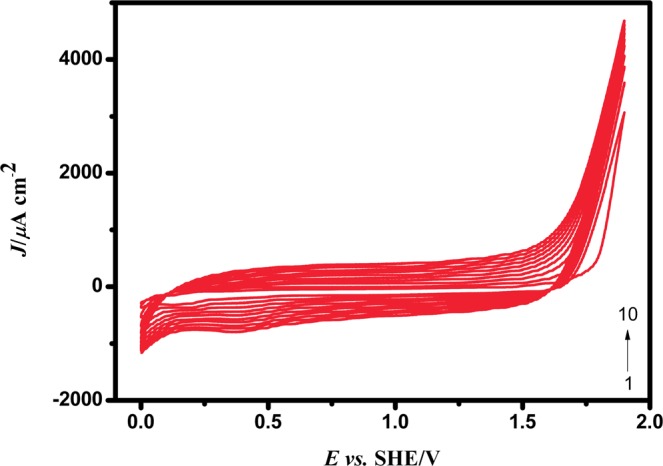
The electrochemical polymerization of EDOT on GCE by 20 mM of EDOT with 50 mM of tetrabutylammonium perchlorate in acetonitrile at a scan rate of 0.05 V/s *vs*. Ag^+^.

A uniform growth of PEDOT on GC surface was formed by cyclic voltammetry during electropolymerization in 10 cycles between the potentials −0.65 V and +1.25 V *vs*. an Ag^+^ pseudo reference electrode. A Pt foil was used as the counter electrode. Figure 7 depicts the uniform deposition of PEDOT formation on clean GC surface. After polymerization, the GC electrode was washed with acetonitrile solution to remove the excess PEDOT or unreacted monomer of PEDOT. A smooth thin film of PEDOT could be formed by this method as reported earlier through other approaches^26,27^.

1 $$m=\frac{({Q}_{dep1})\,{M}_{1}}{F{Z}_{1}}$$

The mass of PEDOT (*m*) was calculated from the total charge of the film after polymerization process, using the amount of charge (*Q*_dep1_), and assuming 100% current efficiency (*η*) (the total charge passed through the cell during the polymer film growth process). *M*_1_ is the molecular weight of EDOT, *F* is the Faraday constant (96,485 Cmol^−1^). *Z*_1_ (=2 here) is the number of electrons transferred^28^. The columbic equivalent for the obtained PEDOT film was found to be 0.040109 *C* and the mass of PEDOT was calculated as 2.974 × 10^−5^g.

#### Electrochemical characterization of Cu-PEDOT

The Cu is potentiostatically deposited on PEDOT in 0.1 M NaClO_4_ containing 2 mM of CuSO_4_ at an applied potential +0.34 V *vs*. SHE for a period of 120 *s* by using chronoamperometric (CA) technique. An abrupt increase in current density value is noticed at the initial stage which attained a steady state around 2 *s*, indicating the nucleation and followed by uniform growth of Cu on PEDOT.

**Figure 8:**
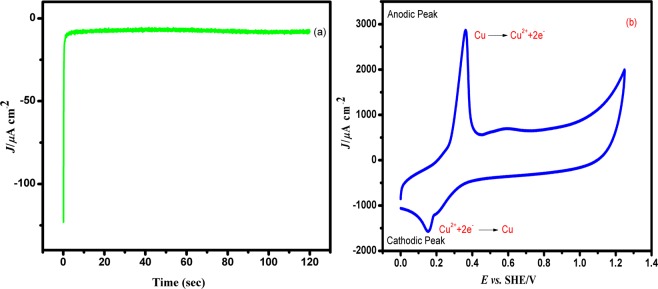
The Cu deposition on PEDOT matrix (**a**) in 2 mM CuSO_4_ with 0.1 M NaClO_4_ at a constant potential of +0.34 V for 120 *s* and (**b**) CV responses for Cu-PEDOT/GC in 0.1 M NaClO_4_ at a scan rate of 0.05 V/s *vs*. SHE.

The controlled potential deposition and formation of Cu on PEDOT has been analysed by cyclic voltammetry which has been shown in Fig. 8a,b respectively. The redox features of Cu appears at 0.36 V on the anodic side and at 0.15 V on the cathodic side. The peak separation between the cathodic and anodic peaks is found to be 0.21 V. The mass of Cu deposited on PEDOT was calculated from cyclic voltammetric data by integrating the charge corresponding to the reduction peak of Cu, and was found to be 4.9354 × 10^−6^ g/cm^2^.

#### Electrochemical characterization of Pd/PEDOT

**Figure 9:**
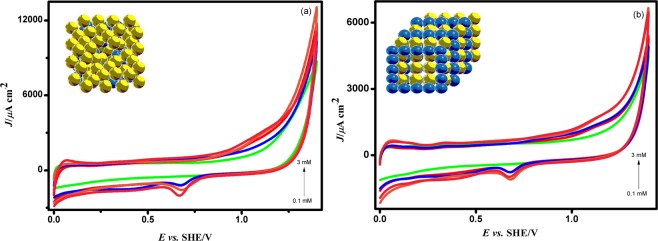
CV responses of Pd/PEDOT (**a**) before and (**b**) after 5 potential cycles in 0.5 M H_2_SO_4_ with varying the concentrations of 0.1, 0.5, 1, 2, and 3 mM of PdCl_2_ in the potential range between 0.0 V and 1.3 V at a scan rate of 0.05 V/s *vs*. SHE.

The Pd/PEDOT/GC was investigated by cyclic voltammetry in 0.5 M H_2_SO_4_ at a scan rate of 0.05 V/*s* and the typical behaviour of Pd is evident in Fig. 9. The characteristic peak corresponding to Pd oxide formation and reduction is seen at 0.90 V and 0.67 V. The HUPD region is not well resolved in Fig. 9a due to the presence of trace amount of Cu on the surface. Hence the Pd/PEDOT electrode was subjected to potential cycling between the 0 V to 0.14 V *vs*. SHE five times in 0.5 M H_2_SO_4_. During cycling, the unreacted Cu will leach out from the surface as shown clearly in Fig. 9b. Pd ions are stable in an acid medium. Here, the Cu will act as a template or sacrificial anode for Pd/PEDOT/GC during cycling. The current peaks appearing between 0 V to 0.14 V *vs*. SHE are due to adsorption and desorption of hydrogen atoms on Pd/PEDOT catalyst in Fig. 9b.

The amount of Pd estimated from Pd/Cu–PEDOT/GC was found to be 0.56550 × 10^−9^g/cm^2^ from the oxide reduction peak and the electrochemical active surface area (ECSA) was calculated based on the charge involved in the monolayer corresponding to oxygen adsorption/desorption on the Pd surface (q_o_) (424 µC cm^−2^)^29^ was 0.00145 cm^2^. The roughness factor (R_f_) of the electrocatalyst has been found to be 0.020546.

2 $$P{d}_{ECSA}=\frac{({Q}_{P{t}^{0}})}{424\,{\mu }C.c{m}^{-2}}$$

where, ${{Q}}_{P{t}^{0}}$ is the charge corresponding to oxide reduction region in coulombs (C), the monolayer charge corresponding to oxygen adsorption/desorption on the Pd surface is 424 µC cm^−2^.

#### ORR activity of Pd/PEDOT electrocatalyst

**Figure 10:**
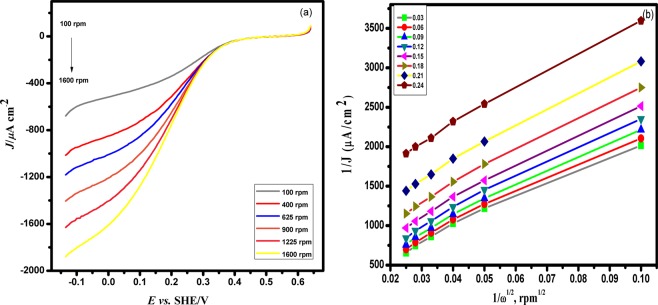
Polarization curve of ORR on (**a**) Pd/PEDOT electrocatalyst in 0.5 M H_2_SO_4_ solution, and (**b**) Koutecky— Levich plots at different potentials.

Oxygen reduction reaction is one of the important factors determining the efficiency of fuel cell cathode catalysts. The Pd/Cu–PEDOT/GC exhibits electrocatalytic properties towards oxygen reduction reaction. Figure 10 a depicts the polarization curves obtained for different rotation rates for Pd/PEDOT catalyst in O_2_–saturated 0.5 M H_2_SO_4_ solution. Obviously, Pd/PEDOT catalyst exhibits a half wave potential at 0.48 V for the ORR, which is nearly 0.3 V more negative as compared with that of the commercial Pd/C catalyst (0.78 V). The Tafel plot of the kinetic currents *j*_k_ of Pd/PEDOT is shown in the Figure S1 (supporting information). In the potential range of 0.5 V > E > 0.4 V, a slop of –105 mV dec^−1^is estimated. The Koutecky—Levich plot (Fig. 10b) showing the plot of inverse current density (j^−1^) as a function of square root of the rotation inverse (ω^−1/2^) and the parallelism and linearity of these plots indicates that the molecular oxygen reduction follows first order kinetics^30^.

3 $$\frac{1}{j}=\frac{1}{{j}_{k}}+\frac{1}{B{\omega }^{1/2}}$$

**Figure 11:**
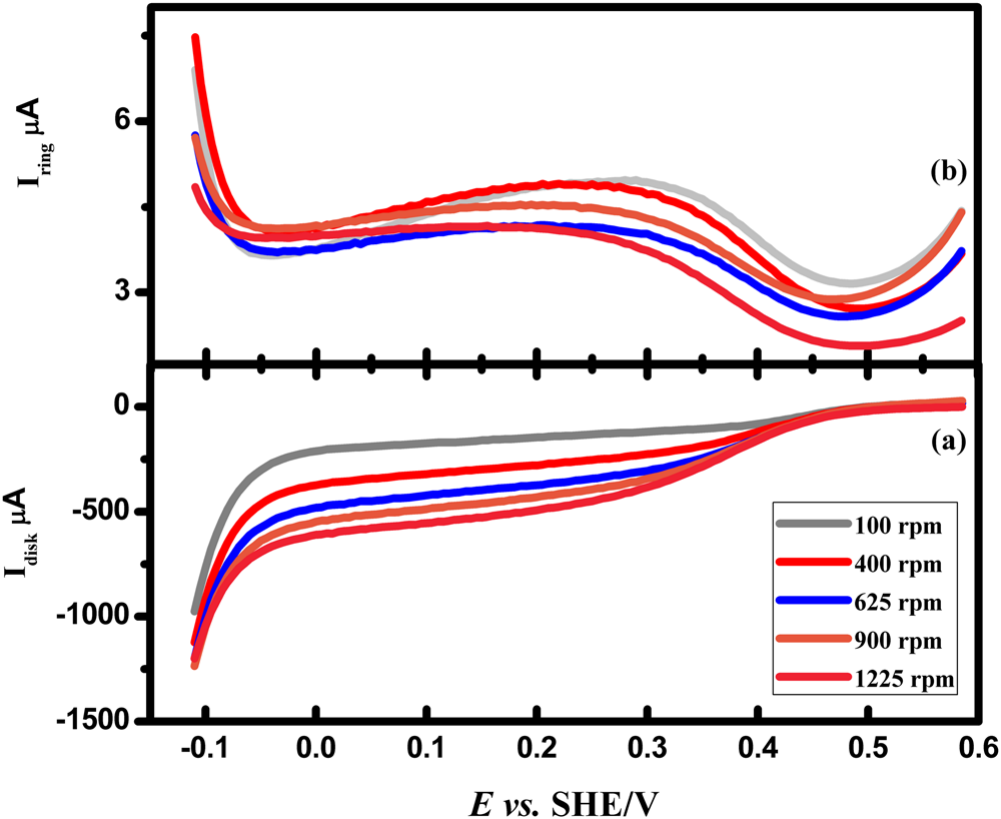
Rotating ring disk electrode (RRDE) measurement of the oxygen reduction reaction (ORR) for Pd/PEDOT electrocatalyst in 0.5 M H_2_SO_4_ solution at a scan rate of 0.02 V/s.

The slope of the K-L plots provides an n value of around 2 (see supporting information for the calculation of electron number). It varies from 2.155 to 2.864 in the range of potentials 0.03 V to 0.24 V. Figure 11 depicts the RRDE measurements for the Pd/PEDOT electrode using the rotation rate which exhibits lower onset potential of oxygen at 0.51 V and half wave potential at 0.48 V *vs*. SHE which are lower compared to other Pt loaded catalyst^31^.

The percentage of the current associated with the peroxide generation at 0.01 V *vs*. SHE has been calculated by using following Eq. 4.

4 $$ \% {H}_{2}{O}_{2}=\frac{2{I}_{R}/N}{{I}_{D}+({I}_{R}/N)}$$

where *I*_*R*_ and *I*_*D*_ are the disk and ring currents and N represent collection efficiency. The percentage of the current associated with the peroxide generation at 0.01 V *vs*. SHE is 8.8%. ORR shows onset around 0.51 V vs. SHE and gradually current increases approximately to 0.2 mA/cm^2^ at 100 rpm. The recorded rpm effect depicts the significant increments in the current with respect to mass transfer process. The proposed mechanism for oxygen reduction is as follows:

5 $${O}_{2}+2{H}^{+}+2{e}^{-}\to {H}_{2}{O}_{2}$$

6 $$2{H}_{2}{O}_{2}\to 2{H}_{2}O+{O}_{2}$$

The low % of H_2_O_2_ indicates that the product H_2_O_2_ obtained by 2 e transfer (Eq. 3) undergoes chemical decomposition to oxygen. Hydrogen evolution follows immediately after oxygen reduction at a potential as low as −0.1 V vs. SHE.

The performance of Pd based catalysts prepared using different supporting matrices have been compared and the results are given in Table 1. The onset potential obtained by us is on par with Pd catalysts prepared using activated carbon supports. It is observed from the table that the inclusion of second metal either as alloy or bimetallic form has shifted the onset potentials to more positive values equivalent to that of Pt/C commercial catalysts. Hence we propose to introduce the second metal also into this Pd/PEDOT matrix by galvanic replacement in future studies.

**Table 1:** Performance comparison of Pd based catalysts on different supporting matrices.

Sl.No	Name of the catalyst	Onset potential (V *vs*. SHE)	Tafel slop value (mV/dec)	Medium	Current density; RDE data (mA/cm^2^)	No. of electrons (n)	Reference
1	Pd/Activated Carbon	0.58	—	0.5 M HClO_4_	1.1*	—	^33^
2	Pd/LaFeO_3_	0.66	92	0.1 M KOH	3.92	2.21	^34^
3	Pd/WC-700-m	0.69	—	0.1 M NaOH	2	3.0	^35^
4	Pt/C (commercial)	1.04					
	PdNi dealloyed	1.04	—	0.1 M HClO_4_	—	—	^36^
	Pd dealloyed	0.90					
5	NM (Nanotubular Mesoporous) -Pd	0.90	67	0.1 M HclO_4_	6.0	4	^37^
	Pd/C	0.95	88		5.0		
6	Pd/PEDOT	0.51	105	0.5 M H_2_SO_4_	1.8	2.8	This work
7	Pd/rGO	0.86	121		—	2.1	^3^
	Pd/PEDOT/rGO	0.98	152	0.1 M KOH	0.82	3.7	

*Taken from CV data at 0.1 V/s.

#### Linear sweep voltammetry studies

**Figure 12:**
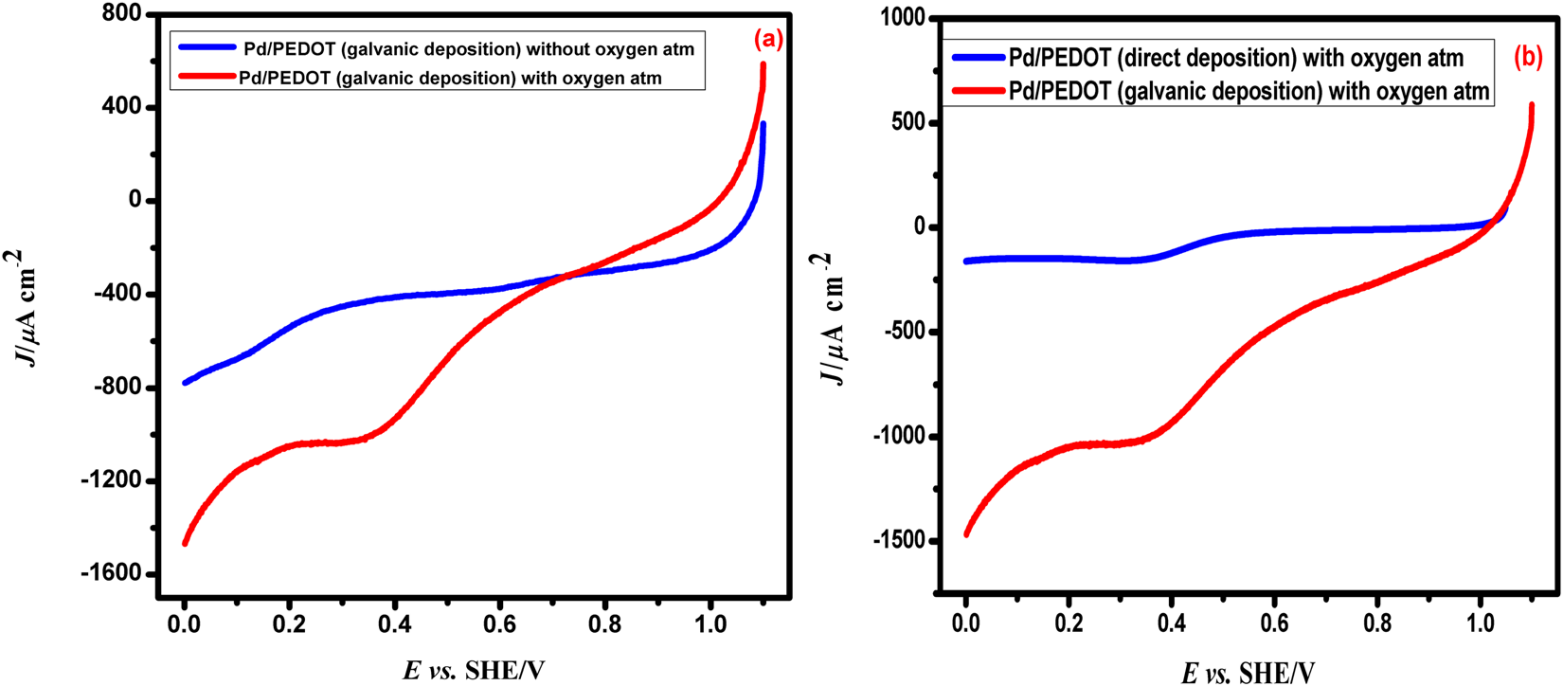
The linear sweep voltammetric response of (**a**) galvanically deposited Pd/PEDOT without oxygen, and with oxygen in a 0.5 M of H_2_SO_4_ solution. Scan rate employed is 0.05 V/s. (**b**) the ORR response of directly deposited Pd/PEDOT and galvanic deposited Pd/PEDOT in 0.5 M of H_2_SO_4_ solution with dissolved oxygen is shown in the figure.

Pd/PEDOT/GC electrode shows a significant enhancement of the cathodic peak indicating the high electrocatalytic activity of the Pd/PEDOT/GC electrocatalyst (Fig. 12a). The directly deposited Pd on PEDOT/GC (control experiment) could show only a lower electrocatalytic activity towards ORR in the absence of Cu template. The galvanically replaced Pd/PEDOT/GC has provided favourable electroactive sites, resulting in more favourable electron transfer kinetics and enhanced electrocatalytic performance towards oxygen reduction in the Pd/PEDOT/GC electrode. Interestingly the galvanically replaced Pd/PEDOT catalyst exhibits the onset potential at 0.728 V *vs*. SHE and the half wave potential occurs at 0.345 V. The mass and specific activities of the Pd/PEDOT catalyst have been calculated as 1.78 A/g and 1009.48 µA/cm^2^.

#### Methanol tolerant reaction for Pd/PEDOT/GC electrocatalyst

**Figure 13:**
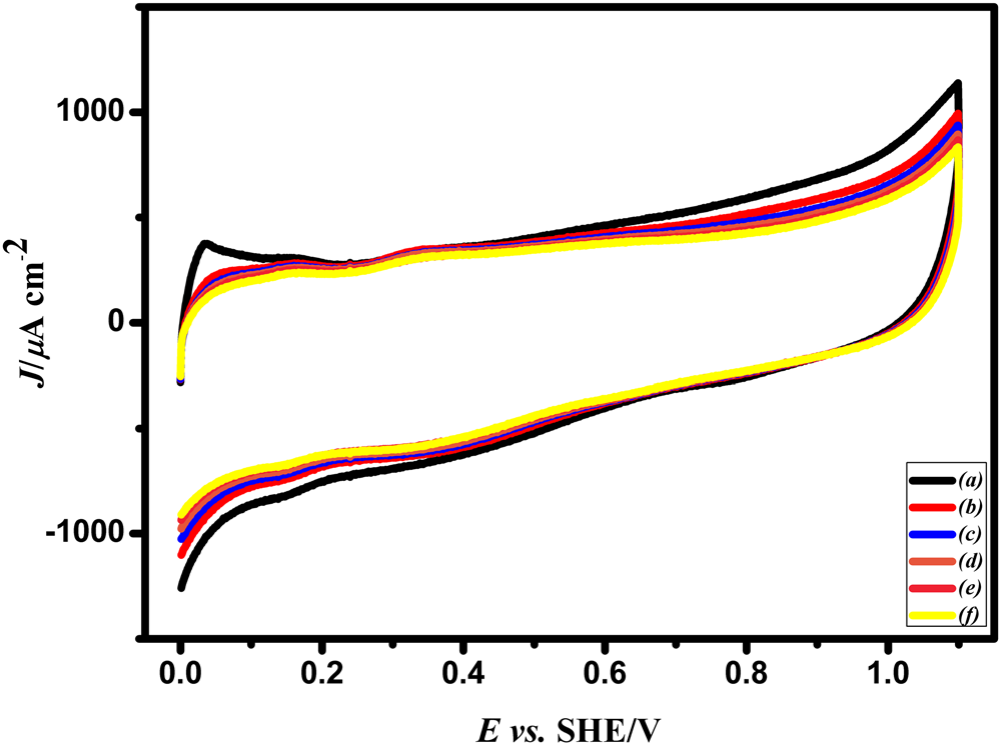
CV responses of (**a**) Pd/PEDOT for successive additions of methanol before, and (**b–f**) after each addition of 1 M CH_3_OH in 0.5 M H_2_SO_4_ in the potential range from 0.0 V to 1.3 V at a scan rate of 0.05 V/s *vs*. SHE.

Currently Pt-containing cathode catalyst does not exhibit much tolerance towards methanol and it leads to decrease in the efficiency of the DMFCs^32^. To investigate such effects, the methanol crossover has been carried out in presence of Pd/PEDOT electrocatalyst in 0.5 M H_2_SO_4_ solution containing 0.5 M to 5 M methanol as depicted in Fig. 13. There is no evidence for methanol oxidation and even at high concentrations of methanol the catalyst current has decreased by 1.212 × 10^−4^A /cm^2^. This might reflect the blocking of adsorbed methanol or some intermediates^30^. In this aspect Pd/PEDOT electrocatalyst might be resolving the problem of methanol crossover in DMFCs. The Figure S2 shows effect of methanol tolerance in presence of oxygen and it indicates facile ORR kinetics in the presence of methanol.

## Conclusion

Pd/PEDOT catalyst was prepared on a glassy carbon surface by initially depositing Cu nanocubes on PEDOT surface under constant potential followed by the formation of Pd on Cu by GDR (galvanic displacement reaction). Superior methanol tolerance property has been observed for this Pd/PEDOT electrocatalyst. This catalyst, favours water formation via hydrogen peroxide production in 0.5 M H_2_SO_4_ with dissolved oxygen. It indicates the superior tolerance for methanol oxidation as well as supports oxygen reduction reaction with 2e^-^ electron transfer yielding H_2_O_2_ which undergoes fast chemical decomposition to water. This paper brings out the catalytic activity of the galvanically replaced Pd nanostructures on PEDOT surface. The morphological analysis carried out during each stage of modification by FE-SEM and TEM analysis, showed a clear transition from porous morphology of PEDOT to nanocube formation of Cu on PEDOT followed by Pd deposit on Cu nanocubes by GDR in the form of uniform agglomeration of Pd nanoparticles with shrimp like structure. Thus prepared electrocatalyst can be used as methanol resistant fuel cell cathode. This concept can open a new avenue to create similar type of catalysts from other Pt group metals with definite structural morphology on polymer matrix for applications in sensing and catalysis using electrochemical approach.

## Supplementary information

Supplementary Information
