# Supplementary material for: Poly (3, 4-ethylene dioxythiophene) Supported Palladium Catalyst prepared by Galvanic Replacement Reaction for Methanol Tolerant Oxygen Reduction
Source: Sci Rep. 2019 Dec 16;9:19184. doi: 10.1038/s41598-019-55688-5 (PMC6915768; doi:10.1038/s41598-019-55688-5)
Supplement: Supplementary file 1 — Supplementary Information [file 41598_2019_55688_MOESM1_ESM.docx]

**Poly (3, 4-ethylene dioxythiophene) Supported Palladium Catalyst prepared by Galvanic Replacement Reaction for Methanol Tolerant Oxygen Reduction**

*Pandia Rajathi. M^a,b^, and Sheela Berchmans^a,b*^*

*^a^EEC Division, CSIR-Central Electrochemical Research Institute*

*Karaikudi 630003, Tamil Nadu, India*

*^b^Academy of scientific and innovative research (AcSIR), New Delhi, India*

**
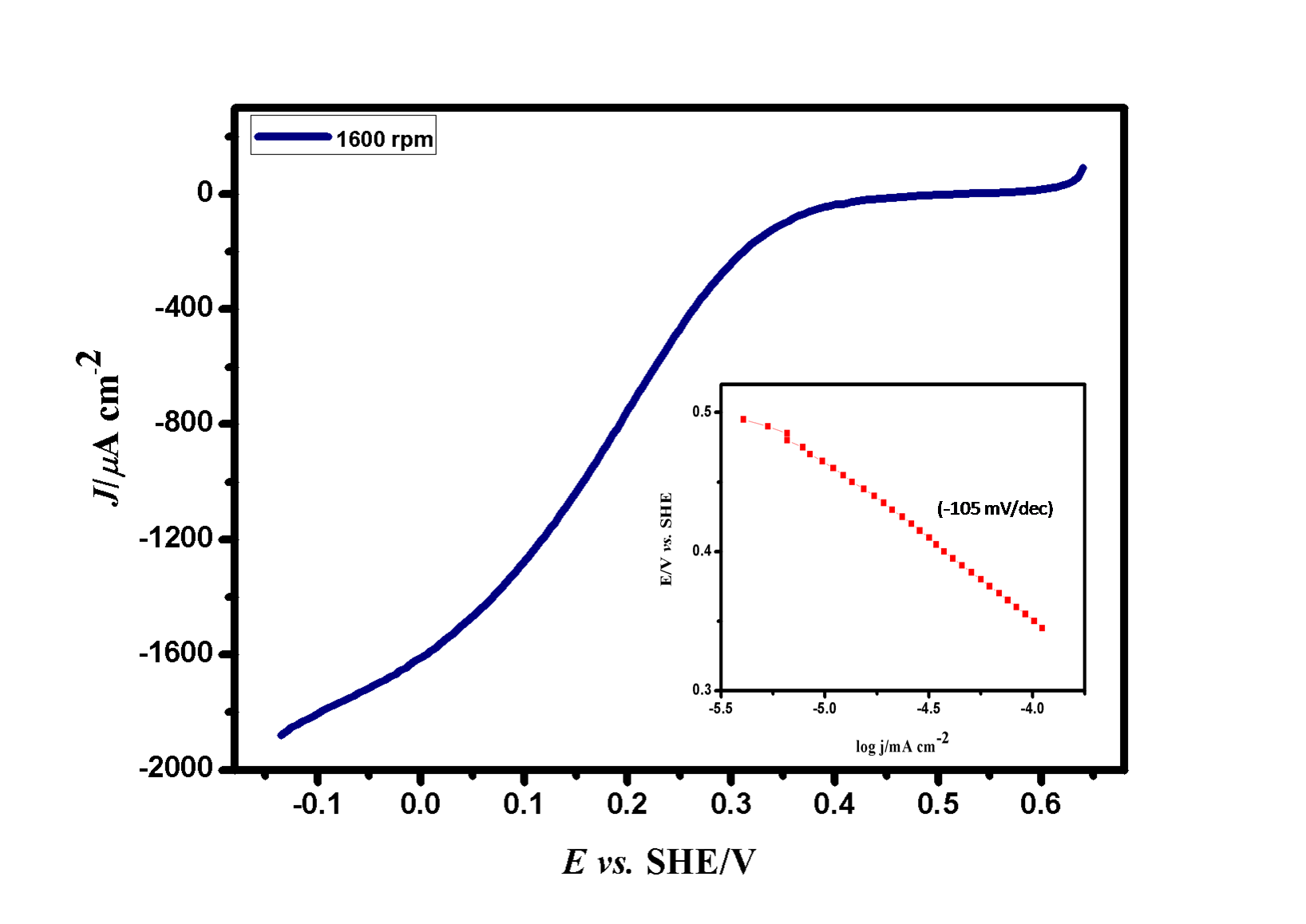
**

**Figure S1: Polarization curve for ORR on Pd/PEDOT electrode 0.5 M H_2_SO_4_ at 1600 rpm. Inset shows the corresponding Tafel plot.**

Calculation of electron number from the kinetic data:

The measured current density is represented as *j*, the kinetic current density is$j_{k}$, ω is the rotation rate and B is a constant. The reported value of the diffusion coefficient of O_2_ (DO_2_= 1.93$\times{10}^{-5}{cm}^{2}s^{-1}$), the concentration of dissolved oxygen in solution ($C_{O_{2}}=1.26\times{10}^{-3}molL^{-1}$), and viscosity of the solution (υ=1.009$\times{10}^{-2}{cm}^{2}s^{-1}$) are used in this equation [1, 2].

$B=0.62nFA{Do}_{2}^{\frac{2}{3}}{}^{\frac{-1}{6}}{Co}_{2}$ (1)

$n=\frac{B}{0.62FA{Do}_{2}^{\frac{2}{3}}{}^{\frac{-1}{6}}{Co}_{2}}$ (2)

Where, F is the faraday constant, and A denotes the geometric area of the electrode.





**Figure S2: Methanol tolerant oxygen reduction in presence of 5 M methanol in 0.5 M H_2_SO_4_ at scan rate of 0.05 V/s**

**References**

[1] Markovic, N. M.; Gasteiger, H. A.; Grgur, B. N.; Ross, P. N. (1999) Oxygen reduction reaction on Pt(111): effects of bromide. J.Electroanal.Chem. 467; 157-163. <https://doi.org/10.1016/S0022-0728(99)00020-0>

[2] Lide, D. R. CRC Handbook of Chemistry and Physics, 75th ed.; CRC Press: Boca Raton, FL, 1995
